# Sexual and Reproductive Health Interventions for Women Exposed to Intimate Partner Violence: A Scoping Review

**DOI:** 10.3390/ijerph22091377

**Published:** 2025-09-02

**Authors:** Leah East, Daniel Terry, Liz Ryan, Brianna Larsen, Amy B. Mullens, Annette Brömdal, Marie Hutchinson, Rebecca M. Jedwab

**Affiliations:** 1 School of Nursing and Midwifery, University of Southern Queensland, Toowoomba, QLD 4350, Australia; daniel.terry@unisq.edu.au (D.T.); liz.ryan@unisq.edu.au (L.R.); marie.hutchinson@scu.edu.au (M.H.);; 2 Centre for Health Research, University of Southern Queensland, Springfield, QLD 4300, Australia; brianna.larsen@unisq.edu.au (B.L.); amy.mullens@unisq.edu.au (A.B.M.); annette.bromdal@unisq.edu.au (A.B.); 3 School of Health, University of New England, Armidale, NSW 2350, Australia; 4 Institute of Health and Wellbeing, Federation University Australia, Ballarat, VIC 3350, Australia; 5 School of Health and Medical Sciences, University of Southern Queensland, Ipswich, QLD 4305, Australia; 6 School of Psychology and Wellbeing, University of Southern Queensland, Toowoomba, QLD 4350, Australia; 7 School of Education, University of Southern Queensland, Springfield, QLD 4300, Australia; 8 School of Health and Health Sciences, Southern Cross University, Coffs Harbour, NSW 2450, Australia

**Keywords:** interventions, intimate partner violence, sexual and reproductive health, trauma-informed care, intersectionality, violence against women, women’s health

## Abstract

Background: Women who experience intimate partner violence (IPV) have a higher likelihood of experiencing detrimental physical, psychological and sexual and reproductive health (SRH) outcomes. However, a gap remains in published literature on SRH interventions available to women experiencing IPV. Methods: A scoping review was undertaken to examine: What is the nature of sexual and reproductive healthcare interventions provided to women experiencing IPV? Five databases (APA PsycInfo, CINAHL Complete, Informit, PubMed and Scopus) were searched on 9 October 2023 for peer-reviewed systematic reviews or primary research published from 1 January 2004–present. The search was repeated on 11 June 2025 to ensure recency of studies. Two researchers independently screened studies at title and abstract, and full-text levels. The two searches yielded a total of 10,844 studies, of which nine were included in the review. Results: Due to the heterogenous nature of the studies, thematic analysis was undertaken with four themes being identified: Outcomes of interventions; Positive impact of personalised and patient-centred care; Professionals’ knowledge, education and training as a facilitator; and Barriers to effective IPV intervention implementation in healthcare. SRH interventions available to women who experienced IPV can empower survivors, improve access to care, and enhance service quality. Conclusions: Evidence-based models of care that are intersectional, trauma-informed and integrated into SRH and IPV services are critical to ensure future work supports women of differing backgrounds who have experienced IPV. Future research should include evaluating effectiveness of interventions, identifying and addressing systemic barriers, and supporting underrepresented groups.

## 1. Background/Introduction

Violence against women is a global healthcare issue resulting in preventable morbidity and mortality [1]. It is well understood that women who experience violence, particularly from an intimate partner, are more likely to experience detrimental physical and psychological health outcomes. In addition to unintended pregnancies and poor mental health, these women are at increased risk of experiencing sexually transmitted infections (STIs), endometriosis, infertility, and miscarriage [1,2]. Despite this evidence, there is paucity of literature focused on the nature of sexual and reproductive health (SRH) interventions provided to women who are exposed to intimate partner violence (IPV).

Although women experiencing IPV have a pronounced need for healthcare, the existing literature indicates that healthcare for exposed women is fragmented, with barriers to access common [3,4]. There is also evidence to suggest that some women choose not to disclose their abuse, with perceived stigma leading women to fear judgement from healthcare providers, which may in turn delay or prevent women from seeking healthcare [3,5,6,7,8]. Additionally, women may be more likely to report past IPV experiences to their healthcare providers when compared to recent or current abuse [9], which impedes the provision of appropriate and timely healthcare interventions. These challenges can result in the misdiagnosis and/or mistreatment of health issues associated with IPV, including associated sexual and reproductive issues [10,11].

Challenges may also present due to the current disconnect between psychosocial and primary care providers for women experiencing IPV. The healthcare needs of women experiencing IPV are often categorised as either psychological (e.g., counselling or social support services) or physical (e.g., emergency department care for physical injuries/ailments), with limited overlap between the two [12,13]. This limited cross-talk between primary care providers and social support services prevents the transfer of relevant health information from one provider to another [4,14], and thus, may hinder appropriate treatment. Moreover, primary care providers that respond to the acute physical health needs of women experiencing violence or abuse (e.g., nurses), often have low awareness of IPV as a health issue and may be unfamiliar with appropriate referral pathways and services [15], especially for the most marginalised and vulnerable survivor populations [16].

Compounding to these barriers, sexual and reproductive healthcare can also be neglected or poorly implemented because discussion surrounding sexual activity concerns can be misunderstood or make health professionals feel uncomfortable, particularly in the absence of appropriate knowledge and/or training [17,18]. It is also worth acknowledging the often stressful and high-stakes nature of working within a healthcare setting, where providers may be time poor, working within an overburdened healthcare system, and have multiple patients simultaneously requiring care. Health worker burnout and compassion fatigue are well documented phenomena (e.g., [19,20,21]) and may manifest as a reduced level of patient care or prioritisation of only the most acute or obvious health concerns. Thus, an additional challenge for women experiencing sexual and/or reproductive ill health due to IPV may be a de-prioritisation of these health concerns by health professionals outside of a family planning environment [22]. Given these barriers, reproductive healthcare providers are uniquely placed to recognise and assist women of differing backgrounds experiencing IPV [23]. To identify current gaps in the treatment options, this scoping review was undertaken to understand the nature of sexual and reproductive healthcare interventions available to women exposed to IPV.

## 2. Materials and Methods

The scoping review was guided by the five-step systematic framework developed by Arksey and O’Malley [24], including the extension developed by Levac et al. [25]. This approach was selected due to the exploratory nature of the research question, the anticipated heterogeneity of study designs and outcomes, and the need to map the breadth of existing evidence rather than assess intervention effectiveness. A scoping review methodology is well-suited to identifying key concepts, types of evidence, and gaps in the literature, thereby aligning with the objective of comprehensively describing SRH interventions for women experiencing IPV.

Within this context, the framework outlines five key steps for conducting a scoping review: (1) Developing the review question; (2) Finding studies; (3) Study selection; (4) Data synthesis; and (5) Data collation, summary and reporting. A search was undertaken on the PROSPERO website to assess whether any existing protocols or reviews had been published on this topic, with no results found. The protocol was not registered or published. The review is reported using the Preferred Reporting Items for Systematic reviews with extension for Scoping Reviews (PRISMA-ScR) checklist [26] (Supplementary Material S1).

### 2.1. Review Question

The question guiding this scoping review was what is the nature of sexual and reproductive healthcare interventions provided to women experiencing IPV?

### 2.2. Search Strategy

A search strategy was developed with guidance from an experienced University healthcare librarian. A broad range of databases were selected to ensure breadth and depth of previously published literature, including APA PsycInfo, CINAHL, Informit, PubMed and Scopus. The search was undertaken on 9 October 2023 and updated on 11 June 2025. Search terms, and eligibility criteria were developed from the review question’s concepts. The search terms, inclusion and exclusion criteria and full search strategy for each database are included in Supplementary Material S2.

### 2.3. Eligibility Criteria

Articles were included if they were peer-reviewed systematic reviews or primary research. The date range of 1 January 2004 onwards was selected due to publication of the first international reproductive health strategy, inclusive of sexual health, and the recognition of the impact of IPV on women’s SRH in this year [27]. Inclusion criteria encompassed interventions focused on sexual and/or reproductive healthcare for women who had experienced IPV. For studies that were focused on violence against women, findings specific to IPV were extracted, including studies within reviews. Woman was defined as cisgender, as identified in each individual study. Studies had to be published in English due to lack of sufficient funding for translation services. Lastly, grey literature was excluded to preserve methodological rigor, and to ensure greater reliability and transparency, particularly in the context of sensitive health interventions.

### 2.4. Screening Procedure

Studies identified through database searches were imported into EndNote. Duplicates were removed and studies were then imported into Covidence. Two members of the research team individually assessed each study at the title and abstract, and full-text levels for eligibility. Screening was completed by four research team members (LE, DT, LR, RJ). The research team met before and during each screening stage and any disagreements were resolved using a fifth researcher (MH).

### 2.5. Quality Assessment

Quality appraisal for quantitative and qualitative studies was completed using the Mixed Methods Appraisal Tool (MMAT) [28], and the Joanna Briggs Institute (JBI) Checklist for Systematic Reviews and Research Syntheses was used for the literature and systematic reviews [29]. Overall, the methodological quality of included studies was mixed. Most met core criteria related to research clarity and appropriate data collection, though several had unclear reporting on appraisal processes and potential bias. These variations should be considered when interpreting the findings (Supplementary Material S3). In accordance with scoping review methodology, variations in quality were noted; however, they did not determine inclusion or interpretation. As such, no studies were excluded based on quality [24,25].

### 2.6. Data Charting and Analysis

Data extraction was completed in Covidence using a data extraction form developed by the research team. The items within the form were iteratively developed to include study characteristics (including the aim, design, participant inclusion and exclusion criteria and total number of participants), participant demographics, type of violence and identification strategy and details about the intervention (including the intervention type, location and referral) and main findings. Two research team members individually performed data extraction for each study (total of three research team members completed data extraction: LE, DT, RJ).

Analysis and synthesis of the study findings included descriptive synthesis due to the limited availability and heterogeneity of quantitative data that precluded the use of meta-analysis, which was also outside of the scope of this review [30]. As such, thematic analysis was undertaken within the context of the review question and any implications for future research, policy and/or practice [31,32,33]. Thematic analysis was undertaken independently by two researchers (LE and DT) allowing for generation and defining of themes. A revised six-step guide was flexibly applied to capture the “uniting idea” for each theme [32].

### 2.7. Ethics Approval

This review synthesises data from previously published studies, therefore no ethical approval was required.

## 3. Results

**Figure 1:**
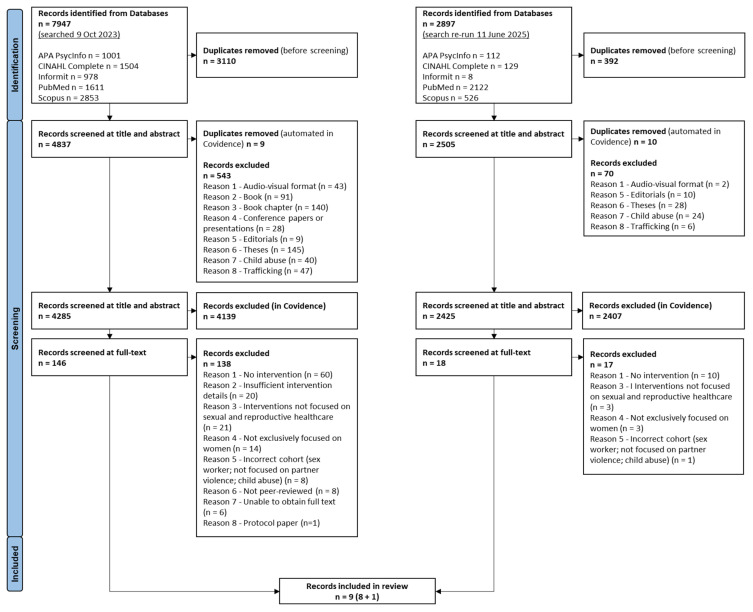
PRISMA diagram.

The five database searches on 9 October 2023 resulted in a total of 7947 records. Following screening, 146 records met eligibility for review at full-text level, of which 138 were excluded. The most common reasons for exclusion included, a lack of an intervention, insufficient detail about the intervention, interventions not focused on sexual and reproductive healthcare, studies not exclusively focused on women, incorrect population cohort, non-peer-reviewed sources, or protocol papers (Figure 1). Eight remaining records were included in the review. The repeated search on 11 June 2025 identified one additional paper to be included. A total of nine papers were included in the final analyses. See Figure 1 for studies included and excluded with rationale at each stage.

### 3.1. Study Characteristics

Three studies provided comparative analyses of interventions using randomised or cluster controls [34,35,36], three were systematic reviews [37,38,39], two were qualitative studies [40,41] and one study utilised a mixed-method approach [42]. Most studies originated from the United States of America (USA) (n = 6), followed by India (n = 1), Kenya (n = 1), and one focusing on multiple countries. Publication dates ranged from 2011 and 2024. Participant numbers ranged from 72 [41] to 12,078 [37]. An overview of study characteristics including aim, design, study inclusion and exclusion criteria, main findings and number of participants is detailed in Table 1.

The focus of the studies included implementing interventions into differing services, outcomes of interventions and possible barriers/facilitators of these interventions [34,35,36,40,41,42], and synthesis of evidence to explore interventions and outcomes in reproductive and sexual health to address violence against women [37,38,39]. When reported, the sociodemographic information varied greatly between studies. Detailed sociodemographic, relationship and intervention information are provided in Table 2, including study intervention details.

### 3.2. Identified Themes

**Figure 2:**
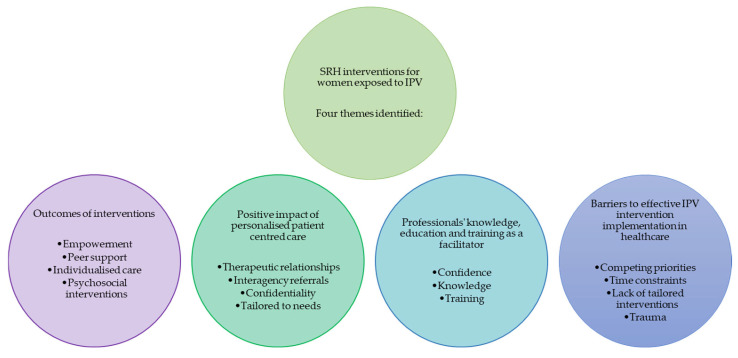
Diagram of identified themes.

Four themes were identified within the literature (Figure 2). The first theme was *Outcomes of interventions* which discusses survivors being able to access peer support and gain a feeling of empowerment and control, and the ability for healthcare workers to provide individualised care. The second theme, *Positive impact of personalised and patient-centred care* details the results of these interventions, bringing about positive change and feelings of safety, respect, and being heard, thus being able to start the healing process. *Professionals’ knowledge, education and training as a facilitator* was the third theme. This theme included the impact of education and training of healthcare professionals being able to implement interventions and supports, with increased knowledge and understanding enabling greater intervention implementation. The final theme, *Barriers to effective IPV intervention implementation in healthcare*, discussed barriers to intervention implementation with influencing factors from consumers, healthcare providers and organisational perspectives. Each of these themes are detailed separately.

### 3.3. Outcomes of Interventions

The studies provided comprehensive insights regarding the various interventions aimed at supporting survivors of IPV, where there is a particular focus on empowerment, peer support, individualised care, and focused psychosocial interventions [39,40,42]. These approaches to care have shown significant outcomes in enhancing the wellbeing and recovery of survivors, such as personal safety, physical health, mental health, and improved quality of life [34,36,38,39,42]. Empowerment was a critical outcome of these interventions, which involved helping survivors regain control over their lives and decisions through skill-building, safety planning, trauma-informed education, and linkage to supportive services tailored to their individual needs. For example, in one study, survivors reported a sense of relief and increased confidence after participating in empowerment-focused interventions. This empowerment was further reinforced by providing information and resources, enabling individuals to make informed decisions about their health and safety [38].

Peer support was identified as another vital feature, with it creating a sense of community and understanding among survivors [35,37]. Participants felt more comfortable and supported when they could share their experiences with others who had similar experiences. Peer support groups also facilitated a sense of belonging and validation, which were essential for emotional recovery. The shared experiences within these groups fostered a supportive environment where survivors could openly discuss their challenges and triumphs [35].

Individualised care and focused psychosocial interventions were also highlighted as significant outcomes. Individualised care involved tailoring interventions to meet the specific needs of each survivor, considering their unique experiences and circumstances [37,38]. This personalised approach ensured that survivors receive the most relevant and effective support. For example, focused psychosocial interventions, such as counselling and therapy, were effective in addressing the psychological impacts of abuse [37,38,39]. The literature indicated that such interventions have been effective in reducing symptoms of trauma, anxiety, and depression among survivors [38]. By providing a safe space for survivors to process their experiences and emotions, these interventions play a crucial role in their overall recovery and wellbeing [38].

### 3.4. Positive Impact of Personalised and Patient-Centred Care

Several positive impacts of therapeutic relationships with healthcare providers and interagency referrals or connections were present in the context of care for survivors of violence. The empathetic approach of healthcare providers, who displayed sensitivity and supportive demeanour, helped the survivors feel more confident and active in their treatment process [42]. Therapeutic relationships fostered a sense of control and empowerment, which was indicated to be crucial for individuals who had experienced IPV including reproductive coercion (RC) [38].

Another positive impact was the role of interagency referrals and connections in providing comprehensive care [36,37,41]. The literature illustrated how effective collaboration between different agencies, such as healthcare providers, sexual health clinics, and advocacy services, can enhance the support system and care for survivors [40]. For instance, the involvement of Sexual Violence Advocates provided additional layers of support [40]. These interagency connections ensured that survivors received not only medical care but also emotional and legal support, which are essential for holistic care. The ability to refer patients to specialised services and ensure follow-up care significantly improved the overall quality of care and support available to survivors [38].

The literature also highlighted the importance of confidentiality in building trust and encouraging survivors to seek help [42]. Many women expressed concerns about confidentiality, fearing that their experiences might be disclosed without their consent. Reinforcing the confidentiality of the services provided and ensuring that survivors’ identities were protected helped alleviate these fears [36]. This assurance of confidentiality was particularly important for those who were hesitant to seek help due to the stigma associated with attending sexual health clinics. By addressing these concerns, healthcare providers were able to create a safe and supportive environment that encouraged more survivors to come forward and access the services they needed [36,42].

Lastly, the positive impact of personalised and patient-centred care was evident in the women’s feedback. The ability to tailor the care to meet individual needs, such as allowing patients to leave the service at any point or come back for follow-up appointments, was highly valued. This flexibility and responsiveness to the patients’ comfort levels and preferences played a crucial role in their healing process [42]. This specialised care provides longer consultation times and a more in-depth understanding of the survivors’ experiences and was shown to be instrumental in helping women feel respected, safe, and ready to begin their healing journey [42]. Further, approaches within some included studies highlight culturally tailored approaches including specific priority sub-groups (e.g., Black, Hispanic, Spanish-speaking participants; [38], [35] and [34], respectively); and evidence of community-led or community embedded aspects of the intervention (e.g., community engagement [37] community resources [38]).

### 3.5. Professionals’ Knowledge, Education and Training as a Facilitator

Multiple studies demonstrated that healthcare professionals’ knowledge, education, and/or training in SRH significantly influenced the implementation and effectiveness of IPV interventions [39,41,42]. Specifically, elements such as safety planning, skill building, psychoeducation, and counselling were identified as critical for improving outcomes in diverse populations [39]. These studies also highlighted healthcare professionals’ increased confidence and knowledge through education and training are linked to better adherence and implementation outcomes [34,36,41,42]. For example, the Kenyan study [36] focused on addressing IPV and RC in reproductive counselling among female family planning patients, found that training healthcare providers in contraceptive counselling related to IPV and RC led to higher contraceptive uptake, greater awareness of IPV services, and a reduction in attitudes justifying RC among female participants [36].

Similarly, an IPV and RC trauma-informed assessment and education intervention in family planning clinics found that women who received the intervention felt more confident in their providers’ responses to IPV and perceived care [42]. Of the 37% of women in this study who had experienced lifetime physical or sexual IPV, over half also experienced RC (68%). Providers reported that the intervention helped them direct care, conduct assessments and screenings, and feel more comfortable with sensitive conversations [42].

In addition, another two studies in the USA found that an IPV and RC intervention decreased pregnancy coercion among women who received the intervention and made them more likely to end unhealthy relationships with the healthcare providers reporting greater confidence in discussing IPV and RC due to increased training and knowledge [34,41]. These findings are similar to others [40], who reported training of personnel better equipped staff to discuss reproductive health and harm reduction strategies for and among women in the USA.

In addition, two of the three reviews also highlighted the importance of training, knowledge, and education for healthcare providers in the context of interventions and care provision [37,39]. One systematic review [38] also emphasised the importance of clinician expertise and experience in achieving positive outcomes for IPV interventions. For example, a review of multicomponent interventions addressing safety and health risks among Black women in the USA, found that clinician expertise was crucial for the success of these interventions, although success was not measured [38]. Additionally, healthcare provider knowledge and training were found to influence provider readiness and confidence in delivering care, particularly in low- and middle-income countries [37]. Considering these studies’ contexts with women from low- and middle-income countries around the world, Black female survivors of violence in the USA, and women survivors in India, a significant finding was that none of the three studies explored the role intersectional-informed IPV interventions and care provisions may have on the healthcare providers’ knowledge, education and training as a facilitator.

### 3.6. Barriers to Effective IPV Intervention Implementation in Healthcare

Included studies identified several barriers related to both healthcare provider and systemic issues that impact the implementation and outcomes of interventions. While education and training of healthcare providers positively influence intervention implementation and outcomes, they can also be identified as a barrier. For example, readiness at both the individual and organisational level, including attitudes towards violence against women among employees, were barriers to intervention implementation and outcomes [37]. In evaluating the feasibility of integrating reproductive health services into IPV and sexual violence programs through healthcare provider training, ref. [40] found that institutional priorities acted as a barrier to the implementation of health services within IPV/sexual violence programs. Another study identified lack of time, and uncertainty among health professionals regarding an appropriate time to raise issues of IPV [41].

One significant barrier highlighted within healthcare provider education and training were the lack of tailored interventions addressing specific needs. A tailored reproductive empowerment contraception counselling intervention significantly increased contraceptive use and awareness of IPV services among patients in Kenya [36]. This finding highlights the importance of specialised training for healthcare providers to improve outcomes. Without such personally [36] and culturally [34,35] tailored interventions, healthcare providers may lack the necessary knowledge and skills to effectively support patients, leading to suboptimal care.

Time constraints and conflicting priorities were also demonstrated to pose significant barriers to effective healthcare provider training and education [41]. Literature highlighted the impact of trauma on both women and providers [38,41]. For women, past trauma can make it difficult to engage with healthcare services, especially if they fear re-traumatisation or judgment. The fear of recalling traumatic events, shame, and fear of welfare can deter women from seeking help or fully disclosing their experiences [41]. For healthcare providers, dealing with trauma cases was highlighted as emotionally taxing, leading to burnout and compassion fatigue [38,41]. This emotional burden can affect healthcare providers’ ability to provide empathetic and effective care.

## 4. Discussion

Due to the continued vast unmet and unaddressed healthcare needs for women who have experienced IPV, particularly in relation to SRH care, this scoping review sought to explore and synthesise the SRH interventions provided to women exposed to IPV on an international scale. Findings from this review highlight a variety of facilitators and barriers to effective IPV intervention implementation in healthcare. This included interventions enabling survivors to access peer support and gain feelings of control and empowerment, and healthcare professionals having greater capacity to provide individualised care [35,37,38,39,40,42]. The findings also identified the positive impact the IPV interventions had on the survivors, bringing about positive change where being heard, feeling safe and respected allowed for the healing process to commence [36,42].

Identified interventions regarding SRH included a range of approaches targeting both healthcare providers and women who have experienced IPV. The main foci were training or education for healthcare providers (e.g., IPV, RC), and education or specialised clinics/supports (e.g., SRH, IPV) for women receiving care [34,36,39,40,41,42]. Additionally, a meaningful subset of studies (more than half) included multiple components of these interventions [34,37,38,39,42]. Further, the current studies identified a variety of interventions provided to women who have experienced IPV, including support groups/clinics focused on IPV and/or reproductive health, education for healthcare providers and women, and interventions targeting priority sub-groups (e.g., Hispanic women or Black women) [35,37,38]. The interventions did not focus on routine screening and assessment for IPV, but focused on training, education, and specialised support. Significantly, the relevant knowledge, education and training-focused studies [37,38,39] overlooked the importance of considering intersectionality and intersectional-informed IPV interventions and care provision on the healthcare providers’ knowledge, education and training; central to women survivors of violence part of multiply marginalised and vulnerable communities [16,48].

Consistent with previous research, interventions that were provided in varied modalities also demonstrated how IPV and SRH can be integrated within existing models of care. This enabled greater feasibility, cost-effectiveness, more seamless access by service users, and protection of privacy/confidentiality for women under the guise of accessing ‘usual’ healthcare [49,50], including within more discrete primary care settings [51]. Further, findings suggest that attempts at integration of IPV with SRH services, and vice versa, can serve as a viable means for providing more coordinated approaches to prevention, screening and specialised supports. Such endeavours offer a more holistic approach to ensuring continuity of care for issues that highly inter-relate (i.e., IPV and SRH). This contrasts with what has historically occurred when supporting women presenting with either of these concerns in isolation. The integration of IPV and SRH services serves to counteract traditional health vs. welfare sector silos [40].

Given the broad and exploratory nature of this scoping review, and without specific a priori research questions, consistent with scoping review approaches, the findings are synthesised in relation to the emergent themes. These themes relate to SRH interventions provided to women who have experienced IPV surrounding barriers, facilitators, outcomes of interventions and positive impacts of interventions reported by survivors, which were identified through this review. Consistent with this approach, previous research has demonstrated the value in exploring barriers and facilitators as an early phase in growing an empirical literature base and to guide future rigour in intervention delivery and evaluation (see [52]).

### 4.1. Outcomes of Interventions

Empirical evidence highlights the positive impact of interventions seeking to improve the social support and mental health of women survivors who are exposed to IPV, such as reduction in experience of IPV or reduction in partner aggression [53]. The identified studies demonstrated how sexual and reproductive healthcare interventions, which focus on empowerment, helped women survivors of IPV to increase their level of confidence. It enabled them to make informed decisions about their safety and health through the provision of information and resources for example [38]. Other research [54], also found improved mental health outcomes in the women survivors of IPV engaging in interventions focusing on empowerment.

Closely aligned, the studies in this review also stressed how psychosocial interventions such as therapy and counselling addressed the psychological and mental health effects of IPV. This included reducing signs of anxiety, depression and trauma among women survivors, including overall recovery and wellbeing [37,38]. As highlighted within the literature ([55,56]), counselling plays a vital role in the recovery of women exposed to and surviving IPV; however, perpetrators are equally in need of counselling to effectively break cycles of IPV within and across generations [55].

When peer-support was part of the intervention, women IPV survivors expressed feeling more comfortable and supported in sharing their experiences, in turn creating a supportive environment [35]. As outlined elsewhere, peer-support plays a vital role in the context of IPV service provision and is conceptualised as a holistic alternative to ‘traditional’ forms of IPV interventions such as personal growth, including that of the peer-support worker [57]. However, the peer-support model has also been presented with challenges, such as lack of role clarity for peer-support workers, and scepticism towards peer-support workers from credentialed professionals [58].

Parallel to this, the identified articles within this scoping review stressed the importance for IPV interventions to be tailored to the unique needs, experiences and circumstances of the women survivors to ensure relevance and efficacy, including socio-economic status, ethnicity, and cultural and linguistic backgrounds [37,38]. The aspect of tailoring IPV interventions to the needs of the survivors, and intersectionality-informed, have been explored elsewhere linked to housing and homeless [59], social contexts and cultural needs of the women survivors [60,61], and included being sensitive and affirming to the survivor’s sexual orientation [62,63].

### 4.2. Positive Impact of Personalised and Patient-Centred Care

Consistent with previous published literature, findings from this review highlight the importance and value that women place on service providers taking humanistic [64], trauma-informed [65], and client-centred [66] approaches when supporting them. These factors are even more important when working with women who are experiencing heightened levels of and multiple forms of vulnerability, including intersectionality, risk and stigma and help to strengthen rapport and facilitate health service engagement, particularly in highly sensitive and stigmatised topics of IPV and SRH [67,68].

Echoing the findings of the current study, the importance of inter-agency collaboration, cross-referral and advocacy are critical ingredients to effectively engage with and support women who have experienced IPV with SRH options and decision making. The following recommendations have been suggested to enhance the feasibility and benefits of such IPV/SRH collaborations, and include formal partnership agreements, cross-training opportunities for staff, and standardised referral protocols [40]. Further recommendations include policy change to sustain such partnerships, which can be enabled through staff training and greater funding [69].

One additional and paramount consideration illuminated in this study, which enabled trust and help seeking for women who have experienced IPV, was the vital role of felt and actual privacy/confidentiality. This is consistent with literature purporting confidentiality as essential for engagement and intervention to be possible [70], particularly due to the psychological effects of IPV and coercive control and the safety concerns for victim-survivors [71,72].

### 4.3. Facilitators

Findings from the current study lend support for the value and benefit of education and training to enhance healthcare providers’ knowledge and confidence, particularly in topics of heightened sensitivity, stigma and risk (e.g., IPV, SRH) taking an intersectional trauma-informed approach [16]. This enhanced knowledge and confidence by healthcare providers has been demonstrated to lead to more effective implementation of IPV and SRH interventions and potentially better client outcomes (see Zachor et al., 2018 [73]). However, results in a 2021 systematic review demonstrate some inconsistent findings, in part, due to the heterogeneity of studies [74]. Consistent with previous research, there are distinct benefits of ensuring an IPV workforce is better equipped to provide education and referral options for SRH, and in turn for clinicians in SRH to better screen for IPV and provide appropriate referrals for specialised IPV support and advocacy services [73]. A recent integrative literature review [75] has also recommended the need for dedicated funding for responding to IPV training and emphasised the importance of continuing professional development. Future healthcare professional training in the combined SRH and IPV space would also benefit from interdisciplinary approaches tailored to cultural and linguistic needs when supporting members of key priority groups (see [76]), in tandem with community-led approaches [77,78], to maximise potential uptake, relevancy and impact.

### 4.4. Barriers

Healthcare providers often exhibit hesitancy in screening for IPV due to a variety of factors. Despite receiving training, some midwives, nurses, and other healthcare professionals remain reluctant to consistently implement routine inquiries embedded within IPV interventions [79]. This hesitancy can stem from personal discomfort, fear of offending patients, or uncertainty about how to handle disclosures of IPV effectively.

Another significant barrier to the effective implementation of IPV interventions was time constraints. Healthcare providers often struggle to find the appropriate time to raise issues of IPV during consultations, especially when there are multiple presenting concerns that need to be addressed. The need to prioritise other urgent health issues can lead to the de-prioritisation of IPV-related matters. Literature has highlighted [41] that lack of time and the challenge of integrating IPV screening into already busy schedules are common hindrances. This indicates a systemic issue within healthcare settings where the structure and demands of clinical practice do not align well with the needs of IPV screening and intervention. Innovative solutions, such as integrating IPV screening into routine health assessments or using digital tools to streamline the process, could be explored to mitigate these time constraints [80]. At the institutional level, several barriers can impede the effective implementation of IPV interventions. For example, research found [40] that institutional priorities often hindered the integration of reproductive health services into IPV programs. As such, these insights highlight the importance of institutional commitment and the need for standardised, evidence-based protocols to ensure consistent and effective care.

Trauma and burnout also play a critical role in hindering effective intervention implementation. Both women experiencing IPV and healthcare providers can be affected by trauma, which poses additional barriers to effective intervention implementation. Past trauma can make it difficult for women to engage with healthcare services, especially if they fear re-traumatisation or judgment [41]. The fear of recalling traumatic events, feelings of shame, and concerns about welfare can deter women from seeking help or fully disclosing their experiences [41]. Healthcare providers dealing with trauma cases can experience emotional exhaustion, leading to burnout and compassion fatigue [81]. This emotional burden can affect their ability to provide empathetic and effective care. Literature has highlighted ([38,41]) dealing with trauma cases is emotionally taxing for providers, which can impact their performance and wellbeing. Addressing these issues requires a dual approach, which encompasses providing trauma-informed care and support for patients and ensuring that healthcare providers have access to resources and support to manage their own emotional wellbeing. As such, training in trauma-informed care, regular debriefing sessions, and access to mental health resources for providers remains essential.

### 4.5. Strengths

This review spanned multiple countries and distilled evidence on what works for women and the mechanisms through which positive benefit can be achieved. These mechanisms provide evidence on how healthcare providers can address service fragmentation and disconnect in a way that minimises stigma and empower women of differing vulnerable backgrounds who experience invisibility or marginalisation within healthcare systems [16]. The findings offer significant potential for replicating effective strategies to improve outcomes and close health service gaps. The findings could guide the development of SRH and IPV best practice guidelines for sexual health and other health services. Such guidelines could support the development of “no wrong door” approaches that would identify and support affected individuals, regardless of their point of access. Sexual health “first aid” training could be provided across sectors to raise awareness of this important issue among IPV survivors and aid in earlier identification and linking in of women for support, and ensuring services are accessible for women.

### 4.6. Limitations and Future Research Directions

There are several limitations to the current scoping review. Firstly, while peer-review ensured rigour, the focus on peer-reviewed and English only potentially narrowed the scope of the findings [82] and limits geographical diversity and generalisability. This restriction of language may have excluded relevant interventions published in other languages, thereby impacting the comprehensiveness and global applicability of the findings. This concentration of research based in the USA may reflect strong funding and established SRH-IPV integration models within that context; however, it raises concerns about transferability to other cultural and healthcare contexts. Also, the varied methodological approaches and lack of heterogeneity and specificity across studies prevented statistical comparisons. The poor specificity of detail in many of the articles, such as lack of detail on who committed the sexual or domestic violence and the relationship(s) to the women means it is possible that studies on violence that were excluded may have also included IPV but were excluded from the review due to minimal reporting. Thirdly, we considered women as a holistic group, and did not specifically distinguish sub-groups. Future research and reviews should explicitly include or report on trans women to ensure their experiences are represented. Similarly, cultural, linguistic, ethnic, and spiritual diversity was not always reported in the studies, meaning it was not possible to explicitly foreground these important contextual factors in SRH interventions for IPV survivors, nor the extent to which interventions were community-led and appropriately culturally tailored. Finally, this review primarily examined SRH interventions for women experiencing IPV but did not consider the nature of their relationship configurations—such as heterosexual, same-sex, polyamorous, or other forms. These limit understandings of how relationship dynamics may influence access to and experiences with care, and collectively highlight key areas for future research, staff training and health promotion efforts.

## 5. Conclusions

There are a variety of SRH interventions available to women who are exposed to IPV, highlighting their potential to empower survivors, improve access to care, and enhance service quality. Interventions that were intersectional, trauma-informed and client-centred were especially effective in fostering trust and engagement. However, gaps remain, including limited representation of diverse populations, relationship types, and community-led initiatives. More research is needed to guide evidence-based SRH care for women affected by IPV. Future research should focus on evaluating intervention effectiveness, addressing systemic barriers such as provider burnout and institutional constraints, and exploring underrepresented groups and grassroots efforts. Collaborative partnerships and sustainable funding are essential to support these initiatives. Developing holistic, evidence-based models of care that integrate SRH and IPV services is a critical next step. Broader societal change is also needed to challenge gendered power dynamics and improve women’s agency in accessing care. Together, these efforts can build a more inclusive and responsive healthcare system for all women affected by IPV.

## Figures and Tables

**Table 1:** Study characteristics.

Author(s) and Year	Title	Country	Aim	Study Design	Inclusion Criteria	Exclusion Criteria	Main Findings	Total Number of Participants
Decker et al., 2017 [42]	Implementing Trauma-Informed Partner Violence Assessment in Family Planning Clinics	USA	To “describe: (1) the uptake and impact of the ARCHES brief IPV/RC assessment and intervention as implemented in family planning clinics, and (2) feasibility and acceptability as experienced by providers and patients alike.”	Sequential mixed-methods	Quantitative: English-speaking women, ages 18–35, were recruited at two family planning health centres (urban vs. suburban) in the Baltimore area from January to April 2014. Qualitative: English-speaking and ages 18–35, patients and providers.	Not stated	65% reported receiving intervention, with 31% receiving both components. Women with history of experiencing IPV had lower belief that providers care about patient safety (78% vs. 92%, *p* = 0.04). Intervention recipients felt supported that healthcare providers were concerned for their safety (91.9% vs. 73.9%; RR 1.22, 95% CI 1.01–1.47), would know what to do if in an unhealthy relationship (90.7% vs. 67.4%; RR 1.35, 95% CI 1.09–1.66, and knew about resources (33.3% vs. 8.0%, RR 4.29, 95% CI 1.05–17.55). Qualitative feedback indicated IPV assessment was viewed as provider concern for recipients, the safety card supported assessment and discussion.	Quantitative—n = 132 (total 146 recruited but 14 excluded due to incomplete intervention) Qualitative—patient interviewed n = 26, providers interviewed n = 9
Gmelin et al., 2018 [40]	Integrating Reproductive Health Services Into Intimate Partner and Sexual Violence Victim Service Programs	USA	To assess “training for victim service agencies on integration of health services” -Feedback on ‘Project Connect’ -Processes of partnership building between IPV/SV agencies and health services following training -Barriers and facilitators to connecting clients to reproductive and sexual health services	Qualitative descriptive—thematic analysis	‘Project Connect’ sites	Not stated	Referral pathways through partnerships enhanced the care women received through enabling private yet direct referral to healthcare services Training led to improved relationships with other health services, and staff feeling empowered to discuss reproductive health topics	Not stated
Lewis et al., 2022 [37]	Interventions in sexual and reproductive health services addressing violence against women in low-income and middle-income countries: a mixed-methods systematic review	Review—multiple	“To synthesise evidence on the effectiveness, cost-effectiveness and barriers to responding to violence against women (VAW) in sexual and reproductive health (SRH) services in low/middle-income countries (LMICs).”	Mixed-methods systematic review	Studies that evaluated Violence against women interventions in SRH services in LMICs, women of reproductive age 15–49 years and recipients of healthcare, healthcare professionals	Children aged < 15 years, no intervention, systematic reviews	Total 26 studies—reported results had variable impact on women’s health and violence reoccurrence. 11 studies specified IPV as the violence type Interventions were grouped as: During routine sexual or reproductive health consultation; During routine consultation plus community engagement; and In addition to routine consultation.	901 HCP 12,078 women
Miller et al., 2011 [34]	A family planning clinic partner violence intervention to reduce risk associated with reproductive coercion	USA	To examine the “efficacy of a family planning clinic-based intervention to address intimate partner violence and reproductive coercion”	Cluster RCT Intervention occurred at visit, follow-up duration 12–24 weeks post baseline.	All English-and Spanish-speaking women (aged 16–29) who were attending family planning clinics were eligible for screening	Not stated	Women who reported IPV in past three months in intervention arm had 71% reduction in the odds of pregnancy coercion at follow-up compared to control clinics participants (0.29, 95% CI 0.09 −0.91). No significant change in past three months IPV reporting at follow-up for women from intervention or control clinics, regardless of baseline status of IPV. Women in intervention arm also reported stopping dating someone (*p* < 0.001) or stopping unhealthy or unsafe relationship (*p* = 0.013) at follow-up (stratified by IPV reported at baseline).	Eligible clients n = 1337. Agreed to participate n = 1207. Control N = 451, intervention N = 453. (90.3% participation rate) n = 906 women completed the baseline and follow-up survey (retention rate 75.1%). Details related to retention rate or management was not provided.
Miller et al., 2017 [41]	Implementation of a Family Planning Clinic-Based Partner Violence and Reproductive Coercion Intervention: Provider and Patient Perspectives	USA	To explore the perceptions of patients and providers who underwent the intervention	Qualitative descriptive study of participants from larger cluster RCT	Providers and administrators: All staff. Patients: Women aged 18 or older	Not stated	Administrators: Found training day feasible and had improved contact with local services. Providers: 11/18 staff believed intervention supported streamlined contraceptive counselling and was useful reminder to assess for partner violence and RC. Patients: Appreciated the information, some shared the information with others, and they felt supported and less isolated.	23 clinic staff (18 providers, 5 administrators) 49 patients (one group)
Mitrani et al., 2017 [35]	Participation in SEPA, a Sexual and Relational Health Intervention for Hispanic Women	USA	To examine participant characteristics that are barriers and facilitators of engagement in the randomized trial conducted with Hispanic women in the United States	RCT (‘Health, Education, Prevention, and Self-Care’ arm)	Self-identified as Hispanic, aged 18–50 years, reporting sexual intercourse in the past 3 months	Not stated	Higher levels of education (B = 0.12, SE = 0.04, *p* = 0.002), IPV (B = 0.62, SE = 0.26, *p* = 0.016), and American acculturation (B = 0.44, SE = 0.16, *p* = 0.006) were associated with participants engaging with the intervention. Physical violence was associated with treatment engagement (χ2(1, N = 274) = 4.23, *p* = 0.040, OR = 2.63).	Control N = 274. Intervention arm N = 274 N = 111 (41%) did not attend any sessions.
Sabri & Gielen, 2019 [38]	Integrated multicomponent interventions for safety and health risks among black female survivors of violence: A systematic review	USA	a) “To describe the characteristics and effectiveness of evidence-based, integrated multicomponent intervention strategies for Black women survivors of violence”; and b) “To determine the efficacy of various integrated multicomponent interventions strategies on the following individual outcomes for survivors of violence: violence reduction, reproductive health, reduced risk of HIV, reduced stress/ stress management, and improved mental health”	Systematic review	Evaluation quantitative studies, Black women > 18 years survivors of violence, multicomponent intervention, published in English	Inventions focused on men, participants <18 years, interventions that only included one component, lacked evaluation, sample not identified and not distinguish black women as part of the sample, not conducted in the USA	Total 16 papers included in review but only one relevant paper that examined reproductive health outcomes for women experiencing IPV [43]. Individualised intervention improved pregnancy outcomes including reducing preterm births (included counselling, information about community resources, management of depression/mood and education on IPV).	Total N = 1044 (Control N = 523, intervention N = 521) [43]
Sabri et al., 2024 [39]	Integrated domestic violence and reproductive health interventions in India: a systematic review	India	To “identify characteristics of existing evidence based integrated domestic violence and reproductive healthcare interventions in India to identify gaps and components of interventions that demonstrate effectiveness for addressing domestic violence.”	Systematic review	(1) Quantitative or mixed-methods studies evaluating integrated DV and family planning or general reproductive health interventions, including women in prenatal, postnatal, and/or postpartum care. This included screening, prevention, and response interventions; (2) Studies using quantitative or mixed methods randomized controlled trials, non-randomized controlled trials, quasi-experimental or pre-post evaluation designs; (3) Studies that included women of ages 15 and older; (4) Studies conducted in India; (5) Studies published in peer-reviewed journals in English language from 2011–2022.	(1) Studies that did not conduct a quantitative or mixed methods evaluation of an integrated DV and family planning or general reproductive health interventions, (2) Stand-alone DV intervention studies that did not have a family planning or reproductive health component. (3) Studies that did not report findings of an evaluation trial using quantitative or mixed methods experimental, quasi-experimental or pre-post designs. (4) Qualitative studies, literature reviews and study protocols. (5) Studies that included participants under the age of 15; (4) Studies conducted outside India; (6) Studies not published in peer-reviewed journals and not published in English language; and (7) Studies published before 2011	Total 13 papers included in review but only four relevant [44,45,46,47]. Counselling in ante-natal care environment (intervention) led to reductions in financial, emotional and physical abuse [44]. Statistically significant reduction in IPV post-counselling (intervention) [45]. Counselling/education sessions (intervention) had decrease in marital sexual coercion. Both intervention and control reported decrease in marital IPV [46,47].	408 women (all intervention) [44] 1136 women (numbers for intervention not stated) [45] Intervention N = 118, Control N = 102 [46,47].
Uysal et al., 2023 [36]	Effects of a clinic-based reproductive empowerment intervention on proximal outcomes of contraceptive use, self-efficacy, attitudes, and awareness and use of survivor services: a cluster-controlled trial in Nairobi, Kenya	Kenya	“To evaluate the effect of a reproductive empowerment contraceptive counselling intervention (ARCHES) adapted to private clinics in Nairobi, Kenya on proximal outcomes of contraceptive use and covert use, self-efficacy, awareness and use of intimate partner violence (IPV) survivor services, and attitudes justifying reproductive coercion (RC) and IPV”	Parallel-group, prospective, non-randomised, cluster-control trial	Interested in receiving family planning services, female, aged 15–49, not pregnant or sterilised, have a male partner (history of having sex with in the last three months), staying in the area for the next six months, have a mobile phone and able to participate in an interview.	Women who took a health survey (past three months)	Women who received contraceptive counselling had higher rates of taking/using a contraceptive (86% vs. 75%, *p* < 0.001), and significantly greater relative increase in IPV awareness from baseline to three-month follow-up (beta 0.84, *p*-value 0.02, 95% CI 0.13, 1.55) and six-month follow-up (beta 0.92, *p*-value 0.05, 95% CI 0, 1.84). Intervention group also had decrease in attitudes justifying RC from baseline to six-month follow-up (beta −0.34, *p*-value 0.03, 95% CI −0.65, −0.04).	Intervention N = 328, control N = 331 (85% of total screened) Follow-up (6 months) approximately 87% intervention and 80% control

RCT = randomised controlled trial; USA = United States of America.

**Table 2:** Study intervention details.

Author(s) and Year	Age	Sociodemographic(s)	Relationship(s)	Intervention Type	Location of Intervention	Referral to Intervention	Violence Type	How Violence was Identified	Other Comments
Decker et al., 2017 [42]	Lifetime experience of IPV: 55.6% aged 31–35, 45.6% aged 21–25, 34.9% aged 26–30, 12% aged 18–20	Lifetime experience of IPV: 44% identified as White, 40% identified as Other, 31.3% identified as Black or African American	Lifetime experience of IPV: Dating one person/in a serious relationship n = 42.1%, Single/dating more than one person 30.4%, Married 30%	Discussion and/or safety card	Family planning health centre	Self-initiated contact with the research team re: project	IPV, sexual, physical, reproductive coercion	Not stated	
Gmelin et al., 2018 [40]	Not stated	Not stated	Not stated	Training leading to discussions with clients (including reproductive healthcare needs), and referral(s) to family planning clinics	‘Project Connect’ sites (6 states in USA)	Not stated	IPV and sexual violence	Not stated	Participant details not provided. Only site leads took part in phone interviews (did not complete the training)
Lewis et al., 2022 [37]	15–49 years	Not stated	Not stated	Complex healthcare interventions	Sexual health consultations	Not stated	All types of violence against women including IPV, domestic violence and abuse, family violence or non-partner sexual violence	Self	
Miller et al., 2011 [34]	45.7% aged 16–20, 31.6% aged 21–24, 22.7% aged 25–29	Identified as Hispanic 37.5%, Non-Hispanic Black 23.6%, White 22.5%, Asian/Pacific Islander/Other 11.3%, Multiracial 5.1% High school graduate 36.7%, Some college or technical school 34.5%, Some high school 18.3%, Less than high school 1.3%, Graduated from college or technical school 9.2%	In a serious relationship 47.2%, Single/Dating more than 1 person 32.2%, Married/Cohabitating 18.3%, Divorced/Separated/Widowed 2.2%	Enhanced IPV screening which focused on: “educating clients about reproductive coercion and the many forms of IPV, specifically ways in which IPV can affect sexual and reproductive health with respect to control of reproductive choices (e.g., birth control use, condom use, pregnancy and timing of pregnancy)”	4 family planning clinics (2 intervention and 2 control)	Not stated	Birth control sabotage, Reproductive coercion, IPV (physical and/or sexual violence)	Self	
Miller et al., 2017 [41]	39% aged 22–26, 33% aged 18–21, 29% aged 27–30	70% identified as White, 20% as African American or Black, 10% as Multiracial or other	Not stated	Staff trained to offer a trauma-informed intervention addressing intimate partner violence and reproductive coercion to all women seeking care (regardless of exposure to violence)	11 family planning clinics—private space	All women seeking care	IPV, RC	Self	
Mitrani et al., 2017 [35]	Mean 37.31 (SD 8.34)	Education (years) mean 13.62 (SD 3.38) Employed n = 92 (34%)	Living with partner n = 181 (66%)	Sexual health group intervention—consisting of five, 2 h sessions delivered to small groups of women	Community sites “easily accessible to the participants”	(a) Community-based social service (English classes, child care, job development and placement, and health education) organization for Hispanics (b) An urban Florida Department of Health site (c) Flyers posted in the community (d) Study participants also referred family members and friends	IPV, emotional, sexual, physical	Self	
Sabri & Gielen, 2019 [38]	18 years and over	Adult black women	Not stated	One paper related to study aims—reproductive health intervention [43]	Not stated	Not stated	IPV	Not stated	Study reported 1. Characteristics and Effectiveness of Evidence-Based, Integrated Multicomponent Intervention Studies 2. Efficacy of Integrated Multicomponent Intervention Strategies 3. interventions that were trauma-focused or included mental health as an outcome 4. interventions that included HIV risk as an outcome 5. interventions that included stress management as an outcome 6. interventions that included reproductive health issues as outcomes
Sabri et al., 2024 [39]	Over 15 years old, not always stated	Variable	Couples	Counselling and education	Antenatal care setting, not specified	Not specified	DV, IPV, sexual, physical, psychological	Not specified	Various counselling and education interventions—individual, household, and community wide
Uysal et al., 2023 [36]	Mean 26.70 (SD 6.63)	n = 160 (48.78%) secondary education level, n = 95 (28.96%) primary education level or less, n = 73 (22.26%) tertiary education level or higher n = 80 (24.39%) Food insecurity past 30 days n = 230 (70.12%) Paid work past year	n = 230 (70.12%) Married	Health provider training to integrate contraceptive counselling strategies—help women control their contraceptive use, pregnancy decisions, experiences of IPV and reproductive coercion	Six private primary-care clinics operated by a Kenya-based non-governmental organisation, Family Health Options of Kenya	Self-selected (offered a women’s health study upon clinic presentation)	IPV, RC	Not specified	

DV = Domestic violence; IPV = Intimate partner violence; RC = Reproductive coercion.

## Data Availability

The original contributions presented in this study are included in the article/Supplementary Material. Further inquiries can be directed to the corresponding author.

## Supplementary Materials

The following supporting information can be downloaded at: https://www.mdpi.com/article/10.3390/ijerph22091377/s1, Supplementary Material S1 PRISMA-ScR Checklist, Supplementary Material S2 Search terms, inclusion and exclusion criteria and database searches, Supplementary Material S3 Included studies’ quality appraisal.
