# Supplementary material for: Sexual and Reproductive Health Interventions for Women Exposed to Intimate Partner Violence: A Scoping Review
Source: Int J Environ Res Public Health. 2025 Sep 2;22(9):1377. doi: 10.3390/ijerph22091377 (PMC12469413; doi:10.3390/ijerph22091377)
Supplement: Supplementary file 1 [file ijerph-22-01377-s001.zip › Supplementary Material S2.pdf]

Supplementary Material S2

Table 1 – Search terms, inclusion criteria and exclusion criteria developed from the review question's concepts

Concept	Search terms	Inclusion criteria	Exclusion criteria
CONCEPT 1 – Women or female	Women<br>OR Female*	• Women<br>• Aged >18 years old	• Studies not exclusively focused on women
CONCEPT 2 – Intimate partner violence	Violence<br>OR Abuse*<br>OR Assault*<br>OR Rape<br>OR "sexual assault"<br>OR "violence against women"	• Focused on violence against women (including domestic violence and abuse, intimate partner violence, family violence, sexual and reproductive violence and coercion)	• Female genital mutilation<br>• Trafficking<br>• Child abuse<br>• Prostitution
CONCEPT 3 – Reproductive / sexual health	"Sexual health"<br>OR "reproductive health"		
CONCEPT 4 – Health services / interventions	Intervention*<br>OR Healthcare<br>OR "health care"<br>OR Treatment*<br>OR Counsel*<br>OR Service*<br>OR Response*<br>OR Care	• Intervention focused on healthcare needs and/or requirements	

Table 2 – Full search strategy for each database

Database	Search term truncation
APA PsycInfo	(TI women OR AB women) OR (TI woman OR AB woman) OR (TI female* OR AB female*) OR MH Female OR MH Women<br>AND<br>(TI violence OR AB violence) OR (TI abuse* OR AB abuse*) OR (TI assault* OR AB assault*) OR (TI rape OR AB rape) OR MH Violence OR MM Domestic violence OR MH Spouse abuse OR MH Gender-based violence OR MH Intimate partner violence OR MH Rape<br>AND<br>(TI sexual health OR AB sexual health) OR (TI reproductive health OR AB reproductive health) OR MH Reproductive health OR MH Sexual health<br>AND<br>(TI Intervention* OR AB Intervention*) OR (TI healthcare OR AB healthcare) OR (TI "health care" OR AB "health care") OR (TI Treatment* OR AB Treatment*) OR (TI Counsel* OR AB Counsel*) OR (TI Service* OR AB Service*) OR (TI Response* OR AB Response*) OR (TI Care OR AB Care) OR MH Reproductive health services
CINAHL	(TI women OR AB women) OR (TI woman OR AB woman) OR (TI female* OR AB female*) OR MH Female OR MH Women<br>AND<br>(TI violence OR AB violence) OR (TI abuse* OR AB abuse*) OR (TI assault* OR AB assault*) OR (TI rape OR AB rape) OR MH Violence OR MM Domestic violence OR MH Spouse abuse OR MH Gender-based violence OR MH Intimate partner violence OR MH Rape<br>AND<br>(TI sexual health OR AB sexual health) OR (TI reproductive health OR AB reproductive health) OR MH Reproductive health OR MH Sexual health<br>AND<br>(TI Intervention* OR AB Intervention*) OR (TI healthcare OR AB healthcare) OR (TI "health care" OR AB "health care") OR (TI Treatment* OR AB Treatment*) OR (TI Counsel* OR AB Counsel*) OR (TI Service* OR AB Service*) OR (TI Response* OR AB Response*) OR (TI Care OR AB Care) OR MH Reproductive health services
Informit	All Fields:women OR All Fields:woman OR All Fields:female*<br>AND<br>All Fields:violence OR All Fields:abuse* OR All Fields:assault* OR All Fields:rape OR All Fields:"sexual assault" OR All Fields:"violence against women" OR All Fields:"domestic violence" OR All Fields:"domestic abuse"<br>AND<br>All Fields:sexual health OR All Fields:reproductive health OR All Fields:Intervention*<br>AND<br>All Fields:healthcare OR All Fields:"health care" OR All Fields:Treatment* OR All Fields:Counsel* OR All Fields:Service* OR All Fields:Response* OR All Fields:Care
PubMed	women[Text Word] OR woman[Text Word] OR female*[Text Word] OR (Female[MeSH Terms]) OR (Women[MeSH Terms])<br>AND

	violence[Text Word] OR abuse*[Text Word] OR assault*[Text Word] OR rape[Text Word] OR (Violence[MeSH Terms]) OR (Domestic violence[MeSH Terms:NoExp]) OR (Spouse abuse[MeSH Terms]) OR (Gender-based violence[MeSH Terms]) OR (Intimate partner violence[MeSH Terms]) OR (Rape[MeSH Terms])<br>AND<br>sexual health[Text Word] OR reproductive health[Text Word]) OR (Reproductive health[MeSH Terms]) OR (Sexual health[MeSH Terms])<br>AND<br>Intervention*[Text Word] OR Healthcare[Text Word] OR "health care"[Text Word] OR Treatment*[Text Word] OR Counsel*[Text Word] OR Service*[Text Word] OR Response*[Text Word] OR Care[Text Word] OR (Reproductive health services[MeSH Terms])
Scopus	women OR woman OR female*<br>AND<br>violence OR abuse* OR assault* OR rape OR “sexual assault” OR “violence against women” OR “domestic violence” OR “domestic abuse”<br>AND<br>“sexual health” OR “reproductive health”<br>AND<br>Intervention* OR healthcare OR “health care” OR Treatment* OR Counsel* OR Service* OR Response* OR Care
