# Supplementary material for: Sexual and Reproductive Health Interventions for Women Exposed to Intimate Partner Violence: A Scoping Review
Source: Int J Environ Res Public Health. 2025 Sep 2;22(9):1377. doi: 10.3390/ijerph22091377 (PMC12469413; doi:10.3390/ijerph22091377)
Supplement: Supplementary file 1 [file ijerph-22-01377-s001.zip › Supplementary Material S3.pdf]

Supplementary Material S3

Table 1 – JBI Critical Appraisal Checklist for Systematic Reviews and Research Syntheses

**Author(s) and year**	1. Is the review question clearly and explicitly stated?	2. Were the inclusion criteria appropriate for the review question?	3. Was the search strategy appropriate?	4. Were the sources and resources used to search for studies adequate?	5. Were the criteria for appraising studies appropriate?	6. Was critical appraisal conducted by two or more reviewers independently?	7. Were there methods to minimize errors in data extraction?	8. Were the methods used to combine studies appropriate?	9. Was the likelihood of publication bias assessed?	10. Were recommendations for policy and/or practice supported by the reported data?	11. Were the specific directives for new research appropriate?
[34]	Yes	Yes	Yes	Yes	Yes	Yes	Unclear	Unclear	Unclear	Yes	Yes
[35]	No	Yes	Yes	Yes	Unclear	Unclear	Unclear	Unclear	Unclear	Yes	Yes
[36]	No	Yes	Yes	Yes	Yes	Yes	Unclear	Unclear	Unclear	Yes	Yes

Table 2 – Mixed Methods Appraisal Tool (MMAT) - Qualitative

Author(s) and year	Are there clear research questions?	Do the collected data allow to address the research questions?	Is the qualitative approach appropriate to answer the research question?	Are the qualitative data collection methods adequate to address the research question?	Are the findings adequately derived from the data?	Is the interpretation of results sufficiently substantiated by data?	Is there coherence between qualitative data sources, collection, analysis and interpretation?
[37]	No	Yes	Yes	Yes	Yes	Yes	Yes
[38]	No	Yes	Yes	Yes	Yes	Yes	Yes

Table 3 – Mixed Methods Appraisal Tool (MMAT) - Quantitative non-randomised

Author(s) and year	Are there clear research questions?	Do the collected data allow to address the research questions?	Is the sampling strategy relevant to address the research question?	Is the sample representative of the target population?	Are the measurements appropriate?	Is the risk of non-response bias low?	Is the statistical analysis appropriate to answer the research question?
[39]	Yes	Yes	Yes	Yes	Yes	Can't tell	Yes
[31]	No	Yes	Yes	Yes	Yes	Yes	Yes
[32]	No	Yes	Yes	Yes	Yes	Yes	Yes
[33]	Yes	Yes	Yes	Yes	Yes	Yes	Yes
